# Epigenetics—New Frontier for Alcohol Research

**DOI:** 10.35946/arcr.v35.1.01

**Published:** 2013

**Authors:** Shivendra D. Shukla, Samir Zakhari

**Affiliations:** **Shivendra D. Shukla, Ph.D.,***is Margaret Proctor Mulligan Professor in the Department of Medical Pharmacology & Physiology, School of Medicine, University of Missouri, Columbia, Missouri.*; **Samir Zakhari, Ph.D.,***former director of the Division of Metabolism and Health Effects at the National Institute on Alcohol Abuse and Alcoholism, is Senior Vice President of Science, the Distilled Spirits Council of the United States (DISCUS), Washington, DC.*

The term “epigenetics” is rapidly becoming one of the more important watchwords in the field of alcohol research. Put simply, epigenetics is the study of changes in gene function that occur without a change in the body’s genetic code, instead relying on epigenetic markers on, among others, the DNA and certain nuclear proteins to turn genes “on” and “off.” Epigenetic changes also are brought about by histone modifications, as well as by the role that noncoding RNA (ncRNA) plays. By acting on these epigenetic markers, environmental factors such as diet, stress, and prenatal nutrition can make an imprint on the genes that are active in different tissues and at various stages of life. Even more importantly, these alterations may be passed along from one generation to the next. The result is that the influences from harmful environmental factors can be extended beyond the individual and passed to his or her offspring.

This issue of *Alcohol Research: Current Reviews* explores the concept of epigenetics and the role it plays—not only in shaping the key characteristics that ensure normal functioning but also which may lead to disease. In particular, the issue looks at how epigenetics influences the body’s response to alcohol and the development of alcohol use disorders and various disease states.

The concept of epigenetics is not new, originating in the 1940s (Waddington 1942). The idea was put forth to explain alterations in an organism’s phenotype that could not be attributed to modifications in its genotype. Or, in more modern terms, epigenetic modifications are the reason that identical DNA sequences can lead to different gene expression profiles. The specific nature of these modifications, however, has come to light only in recent years. These findings have painted a fascinating and complex picture involving the coordinated interplay of numerous regulatory epigenetic mechanisms that help ensure the organism’s normal development and function as well as adaptability to changes in environmental conditions. Importantly, epigenetic modifications of both DNA and histones are time- and tissue- or organ-specific; as a result, disruptions of the epigenome can have vastly diverse consequences, depending on the developmental stage and tissue or organ affected.

One crucial epigenetic mechanism involves methylation of the DNA, particularly in regulatory regions, which typically results in the silencing of genes. Other modifications center on the histone proteins that help package the DNA in the cell nucleus and which determine how accessible the DNA is to the proteins required for gene expression. Finally, several types of non–protein-coding RNA transcripts also can influence the epigenetic status of the cell. If any of these finely tuned mechanisms goes awry, changes in gene expression result that can increase susceptibility to disease (Shukla and colleagues 2008). In fact, epigenetic mechanisms have been linked to numerous diseases, including cancer, autoimmune disease, and age-related and neurological disorders (Moss and Wallrath 2007; Rodenhiser and Mann 2006).

Environmental factors, including toxic agents and drugs, can exert some of their harmful effects by altering normal epigenetic patterns, leading to abnormal expression or silencing of essential genes and their encoded proteins. Alcohol is fast emerging as one of the chief agents to alter the epigenome of cells and tissues throughout the organism.

The precise regulatory mechanisms through which ethanol alters DNA methylation and histone modifications and, consequently, gene expression are only beginning to be elucidated. This issue features some of the latest discoveries in the field. The authors summarize what is currently known about epigenetic changes related to alcohol metabolism and explore the relationship between alcohol-related epigenetic disturbances and in utero development and the pathophysiology of fetal alcohol spectrum disorders (FASD). Other reviews demonstrate how far-reaching epigenetic influences can be, influencing all major body systems, including the liver and gastrointestinal system, the brain, and the immune system.

Clearly, epigenetic changes, whether transient or permanent, play a pivotal role in mediating alcohol’s actions in a variety of cells and organ systems. Understanding the exact nature of alcohol’s interactions with the epigenome will help scientists design better medications to treat or alleviate a wide range of alcohol-related disorders, including FASD, alcohol addiction, and organ damage. The articles in this journal issue are testament to the progress researchers have made in recent years toward this goal.
